# A Kind of Joint Routing and Resource Allocation Scheme Based on Prioritized Memories-Deep Q Network for Cognitive Radio Ad Hoc Networks

**DOI:** 10.3390/s18072119

**Published:** 2018-07-02

**Authors:** Yihang Du, Fan Zhang, Lei Xue

**Affiliations:** 1 Electronic Countermeasure Institute, National University of Defense Technology, Shushan District, Hefei 230000, China; yuri_wolfdyh@163.com (Y.D.); xwdxh1965@163.com (L.X.); 2 Science and Technology Research Bureau of Anhui Xinhua University, Shushan District, Hefei 230000, China

**Keywords:** cognitive radio, joint routing and resource allocation, responsibility rating, Prioritized Memories Deep Q-Network

## Abstract

Cognitive Radio (CR) is a promising technology to overcome spectrum scarcity, which currently faces lots of unsolved problems. One of the critical challenges for setting up such systems is how to coordinate multiple protocol layers such as routing and spectrum access in a partially observable environment. In this paper, a deep reinforcement learning approach is adopted for solving above problem. Firstly, for the purpose of compressing huge action space in the cross-layer design problem, a novel concept named responsibility rating is introduced to help decide the transmission power of every Secondary User (SU). In order to deal with problem of dimension curse while reducing replay memory, the Prioritized Memories Deep Q-Network (PM-DQN) is proposed. Furthermore, PM-DQN is applied to solve the joint routing and resource allocation problem in cognitive radio ad hoc network for minimizing the transmission delay and power consumption. Simulation results illustrates that our proposed algorithm can reduce the end-to-end delay, packet loss ratio and estimation error while achieving higher energy efficiency compared with traditional algorithm.

## 1. Introduction

With the explosion of wireless communication devices, the spectrum resource has become scarce and crowded. The fixed spectrum allocation policy has resulted in vastly underutilized spectrum holes, which causes the contradiction between the spectrum scarcity and the inefficiency in its usage. Efficient exploitation of the spectrum necessitates Dynamic Spectrum Access (DSA) paradigm. DSA is a supplement and improvement of the existing policy and it can bridge the enormous gulf in time and space between the regulation and the potential spectrum efficiency [1]. CR is a promising technique that adopts DSA paradigm to enhance spectrum utilization, and it allows secondary users (SUs) to access the licensed frequency bands opportunistically in a dynamic and non-interfering manner.

Recently, the research community has aroused interests in Cognitive Radio Networks (CRN) with multi-hop communication requirements (i.e., cognitive radio ad hoc networks and cognitive wireless mesh networks) [2,3,4]. However, one of the critical challenges for operating such systems is how to coordinate the behavior of the upper layer like routing with MAC or physical layer such as spectrum access and power allocation in an incompletely observable and non-stationary environment [5,6]. The location independence in a multi-hop path results in that the available spectrum bands may different at each relay nodes [7]. Moreover, the unavailable channel statistics and unreliable knowledge of topology make it extremely challenging to design an efficient and robust spectrum-aware routing strategy in CRN.

Traditional schemes used to formulate the joint routing and resource allocation problem as an optimization problem, and then to relax it into a distributed one with heuristic solutions for lower computation complexity. An opportunistic cognitive routing protocol was proposed in [8]. A metric named cognitive transport throughput was introduced to evaluate the potential relay gain and a heuristic algorithm was designed to reduce the searching space. The resource allocation problem in cellular networks is studied by jointly employing CR technology and Device-to-Device (D2D) communication in [9]. A two-stage approach is considered to allocate the radio resources efficiently, which consists of an adaptive subcarrier allocation scheme and a novel power allocation scheme. In [10], the system was analyzed from a queuing theory perspective and the cross layer design was formulated as a non-linear integer programming problem. Then a distributed solution was developed based on Lagrangian dual problem. A Routing and Spectrum Allocation (ROSA) algorithm was proposed in [7]. ROSA aimed at maximizing the network throughput without generating harmful interference to the primary users (PUs). The author in [11] presented a formulation of the routing and resource allocation problem with mutual information accumulation. An efficient distributed algorithm was introduced which can minimize end-to-end delay. Traditional schemes attempt to find a fixed path from source to destination [12]. Nevertheless, one of the main weaknesses is that they need priori information such as channel statistics and knowledge of topology which is unavailable in partially observable environment. In addition, fixed routing strategies may fail to address the uncertainties resulted from the dynamic and unpredictable nature of the radio spectrum.

Various learning-based methods have been proposed to adapt the high fluctuation of channel availability in CRN. The author in [13] proposed a distributed Learning Automata for spectrum management problem without any priori information about PUs and other SUs. In [14], a distributed Inter-Cell Interference Coordination (ICIC) accelerated Q-learning algorithm was proposed for robust cognitive spectrum management in LTE cellular systems using the framework of heuristically accelerated reinforcement learning. References [13,14] adopted machine learning approaches to overcome the uncertainty of radio spectrum, but they did not take account routing in multi-hop networks. In [15], the author skillfully combined routing with Q-learning and proposed the Cognitive Radio Q-routing scheme which found least-cost routes as well as its operating channel. The algorithm described in [16] solved the routing problem in CRN with the help of a clustering mechanism reinforcement learning to enhance networks scalability and stability. An online opportunistic routing algorithm in [17] adopted multi-agent reinforcement learning which jointly addressed link and relay selection. Summarizing, the works that adapt Q-learning can only solve the routing problem with limit number of nodes because large network scale will result in the curse of dimensionality. Furthermore, these works solved the joint routing and spectrum access problem without considering transmit power allocation of each nodes, which had effect on energy efficiency and next relay selection. It is believed that transmit power control has to be taken into consideration in order to minimize transmission delay while guaranteeing the energy efficiency.

In this paper, the joint routing and resource allocation problem for cognitive radio ad hoc networks is studied. A variant Deep Q-learning named PM-DQN is adopted to minimize the end-to-end delay of SUs while guaranteeing the energy efficiency and reducing the communication overhead. Our characteristic work can be summarized as follows:

(i) To the best of our knowledge, this is the first work that adopts deep reinforcement learning to solve the routing and resource allocation problem in the multi-hop CRN. It is accepted that deep reinforcement learning can successfully deal with problems with large state space. That is, our method is capable of addressing problems of large-scale networks, which has great advantages compared with the previous work that may hard to converge in the same case.
(ii) In order to guarantee the energy efficiency and control harmful interference to PUs, transmission power allocation is taken into consideration. A novel metric named responsibility rating is introduced, which indicates how much effort SUs should make currently to be responsible for the quality-of-service. The responsibility rating is adopted to help decide the transmission power of every SU node.
(iii) Deep Q-Network uses experience replay to break the strong correlations between samples. However, maintaining large replay memories will increase the overhead and memory occupancy in the communication process. PM-DQN is proposed to reduce replay memory while guaranteeing the system performance. By erasing the inessential transitions, the total memory size can be reduced, i.e., the communication overhead will be lower. In addition, redundancy is also reduced so that learning performance will be enhanced.
(iv) PM-DQN is applied to the routing and resource allocation problem to minimize the transmission delay while guaranteeing the power efficiency. Simulation results illustrates that the proposed scheme outperforms the traditional algorithm in terms of end-to-end delay, power efficiency, packet loss ratio and estimation error.

The mainframe of this paper is organized as follows: the background of reinforcement learning is introduced briefly in Section 2. The system model is described in Section 3. Section 4 introduces responsibility rating and models the routing problem as a Markov Decision Process (MDP). The PM-DQN based joint routing and resource allocation scheme is proposed in Section 5. Section 6 shows the simulation results and Section 7 concludes the paper.

## 2. Background on Reinforcement Learning

For the convenience of the reader, in this section, a brief introduction of reinforcement learning and Deep Q-Network (DQN) is given. This section is far from being an exhaustive survey, and its objective is just to provide the reader with basic information to understand the succeeding sections.

### 2.1. Reinforcement Learning

**Figure 1:**
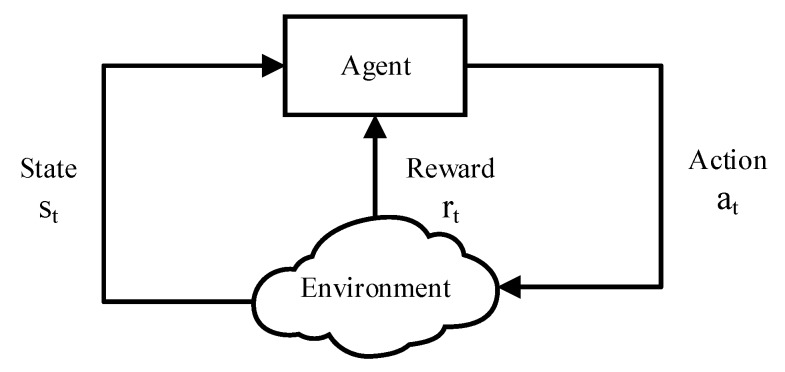
Learning-environment interaction.

Learning can be broadly categorized as supervised, unsupervised and reinforcement learning. Reinforcement learning (RL) is a model-free strategy learning process which aims at learning to act appropriately by interacting with the environment only through trial and error [18]. In general, the RL problem is typically formulated as a finite-state MDP which comprises a discrete state space S and a discrete action space A. Time is represented by a sequence of time steps t=0,1,…. At each time step *t*, the controller observes the system’s current state $s_{t}=s\in S$ and selects an action $a_{t}=a\in A$ to execute. After the action is performed, the state of system transits to a new state $s_{t+1}=s^{\prime }\in S$ with the state transition probability $P_{ss^{\prime }}(a)$. As a result, the environment generates a reward $r_{t}=r(s_{t},a)\in R$ passing to the agent. To understand it better, a graphic representation is shown in Figure 1.

RL approach attempts to find a policy that maximizes the total expected discounted reward over infinite steps. The objective of the agent is then to learn an optimal policy $\pi^{*}(s)$ for each state *s*, in this case, there exits an optimal deterministic stationary policy [19].

The value function of state *s* is defined as the expected infinite discounted sum of rewards that the agent gains if it stars in that state:

(1) $$V_{\pi }(s)=E\left[\sum_{t=0}^{\infty }\gamma r(s_{t},\pi (s_{t}))|s_{0}=s\right]$$

where $s_{0}$ is the initial state, γ∈[0,1) is the discount factor. According to the principle of Bellman’s expectation equation, we can rewrite (1) as:

$$V_{\pi }(s)=E\left[r(s,\pi (s))\right]+\gamma \sum_{s^{\prime }\in S}P_{ss^{\prime }}(\pi (s))V_{\pi }(s^{\prime })$$

It has been proven that the optimal policy satisfies the Bellman’s optimality equation:

(2) $$V^{*}(s)=\underset{a\in A}{\max}\left\{E\left[r(s,a)\right]+\gamma \sum_{s^{\prime }\in S}P_{ss^{\prime }}(a)V^{*}(s^{\prime })\right\}$$

The optimal action-value function is defined as the right-hand side of (3):

(3) $$Q^{*}(s,a)=Q^{\pi^{*}}(s,a)=E\left[r(s,a)\right]+\gamma \sum_{s^{\prime }\in S}P_{ss^{\prime }}(a)V^{*}(s^{\prime })$$

Given the optimal action-value function, we can specify the optimal policy as:

(4) $$\pi^{*}(s)=\arg\underset{a}{\max}\;Q^{*}(s,a)$$

### 2.2. Deep Q-Network

The basic idea of many reinforcement learning algorithms is to estimate the action-value function and choose the state-action pair with the highest Q-value greedily by maintaining a Q-table. Nevertheless, it confronts two challengeable issues when the state space is excessively large. First, the scale of Q-table is too enormous to store and search. Second, the sample is so sparse that the tabular based algorithm (i.e., Q-learning) does not converge. 

In order to overcome the above defects, a non-linear function approximator is used to estimate the action-value function. An approximate value function $Q(s,a;\theta_{i})$ is parameterized using a neural network [20]. A Q-Network can be trained by minimizing a sequence of loss function $L_{i}(\theta_{i})$ that changes at each iteration i:

(5) $$L_{i}(\theta_{i})=E_{s,a\sim \rho (\cdot )}\left[{\left(r+\gamma \underset{a^{\prime }}{\max Q(s^{\prime },a^{\prime };\theta_{i}^{-})-Q(s,a;\theta_{i})}\right)}^{2}\right]$$

where ρ(s,a) is a probability distribution over sequence s and a, γ is the discount factor determining the agent’s horizon, $\theta_{i}$ are the parameters of the Q-Network at iteration i and $\theta_{i}^{-}$ are the parameters used to compute the target at iteration i.

Differentiating the loss function with respect to $\theta_{i}$, the following gradient can be obtained:

(6) $$\nabla_{\theta_{i}}L_{i}(\theta_{i})=E_{s,a\sim \rho (\cdot )}\left[\left(r+\gamma \underset{a^{\prime }}{\max Q(s^{\prime },a^{\prime };\theta_{i}^{-})-Q(s,a;\theta_{i})}\right)\nabla_{\theta_{i}}Q(s,a;\theta_{i})\right]$$

To reduce the computation complexity, the loss function is optimized by stochastic gradient descent frequently. However, the RL approach is known to be unstable or even to diverge when a nonlinear function approximator is used to represent the Q function [21]. The experience replay and fixed parameters of the target network are applied to address the instability in the Deep Q-learning.

## 3. System Model

**Figure 2:**
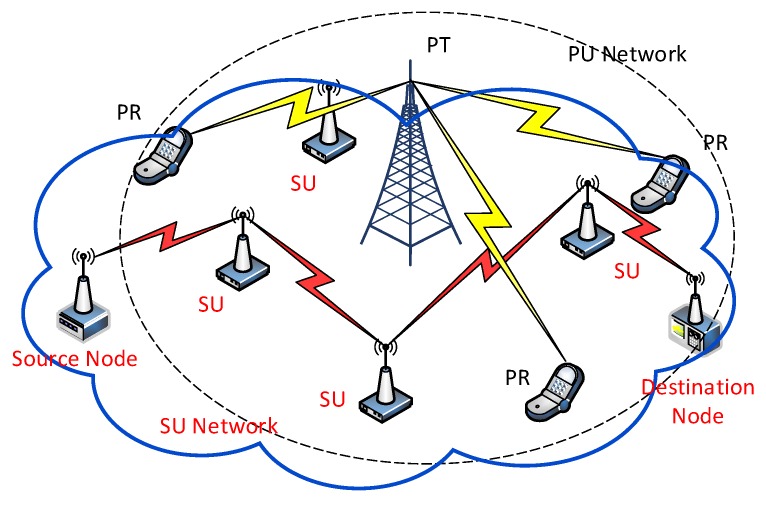
Cognitive radio ad hoc networking scenarios (PT: primary user transmitter, PR: primary user receiver).

We consider a cognitive radio ad hoc network consisting of M PUs and N SU nodes as shown in Figure 2. PUs occupy the specific authorized channels completely and only hold their assigned portion of spectrum. SUs have no licenses for any spectrum bands and opportunistically transmit data in the idle time of PUs. Let (i,j) denotes a unidirectional wireless link from node i to node j, where 0≤i,j<M,i≠j.

The available spectrum can be divided into two categories. The data transmission channel (DTC) is for data communication. It consists of a set of orthogonal spectrum bands $C=\left\{c_{1},c_{2},\ldots ,c_{m}\right\}$, and the available DTC of $SU_{i}$ is represented as $C_{i}$. The common control channel (CCC) is used by SUs to exchange the negotiation. Each SU is equipped with a scanner, a half-duplex normal radio for negotiation transmission and a half-duplex cognitive radio which can reconfigure among C for data communication.

The occupation duration of PUs in each spectrum bands is modeled as an independent and identically distributed alternating ON (when the channel is busy) and OFF (when the channel is idle) process [4]. If the ON and OFF periods of a PU are exponential distribution, and the arrival rate and departure rate of the PU are $\theta_{a}$ and $\theta_{d}$, then the probability of the PU channel being busy is denoted by:

(7) $$P_{on}=\theta_{a}/\left(\theta_{a}+\theta_{d}\right)$$

Apparently, the probability of the PU channel being idle can be represented as:

(8) $$P_{off}=\theta_{d}/\left(\theta_{a}+\theta_{d}\right)$$

The Probability Density Function (PDF) of the OFF periods is given by:

(9) $$f\left(\tau \right)=\left\{ \begin{array}{cc} \theta_{d}e^{-\theta_{d}\tau } & \tau \geq 0\\ 0 & \tau <0 \end{array}\right.$$

Therefore, the probability of the PU being in ON state is given by:

(10) $$\begin{array}{cc} P_{collision} & =1-P\left(t\geq \tau \right)\\ & =1-\int_{\tau }^{\infty }f\left(t\right)dt\\ & =1-e^{-\theta_{d}\tau } \end{array}$$

The PU mean departure rate $\theta_{d}(\mu ,\sigma )$ is computed using the transform in [22] which has an expected mean μ and deviation σ. SUs track the channel statistic through sensing and learning the spectrum usage pattern. Generally, the channel statistic changes slowly and the parameter evaluation can be found in [23,24]. With some localization devices, SUs can only acquire their own location information.

## 4. Problem Formulation for Cross-Layer Design

In order to improve the energy efficiency and control the harmful interference to PUs, power allocation is taken into consideration. In this section, the cross-layer design problem is modeled as a model-free strategy learning process. Firstly, a novel metric named responsibility rating is introduced to make decision on transmission power control. Then the joint design problem is modeled as a MDP, and a deep reinforcement learning approach is adopted to solve the MDP in the next part.

### 4.1. Responsibility Rating

In this paper, an efficient cross-layer scheme is taken account, which jointly addresses routing, spectrum access and power assignment problem in the multi-hop CRN. Conventional RL methods, i.e., tabular based algorithms, behave poorly when dealing with problem with large state space because of curse of dimensionality. Moreover, it is unrealistic to maintain a large lookup table in every relay node. So it is inclined to adopt DQN to solve the joint design problem with large state space.

For energy efficiency and PU protection, we take power adaption into consideration. In this case, the actions of the agent comprise relay selection, channel usage and power allocation, which may result in a huge action space. Though DQN has the capacity of handling problem with large state space, it is not adept in managing problem with huge action space. This is because DQN adopts the Q-learning update framework as the following:

(11) $$\begin{array}{cc} Q_{t+1}(s,a) & =(1-\alpha )Q_{t}(s,a)+\alpha \left[r_{t}+\beta \underset{b\in A}{\max}Q_{t}(s^{\prime },b)\right]\\ & =Q_{t}(s,a)+\alpha \left[r_{t}+\beta \underset{b\in A}{\max}Q_{t}(s^{\prime },b)-Q_{t}(s,a)\right] \end{array}$$

where α∈[0,1) is the learning rate, and β is the discount factor determining the agent’s horizon. The Deep Q-Network is trained by minimizing a sequence of loss function:

(12) $$L_{i}(\theta_{i})=E_{s,a\sim \rho (\cdot )}\left[{\left(r+\beta \underset{a^{\prime }\in A}{\max}Q(s^{\prime },a^{\prime };\theta_{i}^{-})-Q(s,a;\theta_{i})\right)}^{2}\right]$$

We can see that both DQN and Q-learning aim to update the Q-value in the direction of $r_{t}+\beta \underset{b\in A}{\max}Q_{t}(s^{\prime },b)$. And the update of two algorithms needs to search the maximum of $Q_{t}(s^{\prime },b)$, so that we can say DQN adopts the Q-learning framework indeed. That is, the agent has to choose the action with the largest Q function among all actions available. The maximum calculation will have intensive complexity if the action space is particularly large. To handle this problem, a novel concept named responsibility rating is introduced. The transmission time of the SU packet in node i is given by:

(13) $$\tau_{i}=R_{packet}/W\log_{2}\left(1+\frac{h_{ijc}p_{i}}{\sigma +\phi_{ijc}^{PU}}\right)$$

where $R_{packet}$ is the size of SU packet transmitted from source to destination, $h_{ijc}$ is the channel gain between the transmitter node i and the receiver node j, $\phi_{ijc}^{PU}$ denotes the PU-to-SU interference at the receiver node i, and σ is the AWGN power.

If much time is wasted in one hop transmission, it is reasonable that SU node has to increase the transmission power in the next hop in order to reduce the end-to-end delay. In addition, if the delay of one hop is particularly low, then the transmission power can be reduced in the next hop to cut down on power consumption. According to this, the responsibility rating of $SU_{j}$ is defined as:

(14) $$\psi_{j}=\left\{ \begin{array}{ll} \psi_{i}+1 & \tau_{i}>\tau^{*}\\ \psi_{i}-1 & \tau_{i}\leq \tau^{*} \end{array}\right.$$

where $\psi_{i}$ is a nonnegative integer and represents the responsibility rating of $SU_{i}$. In addition, if $\psi_{i}=\max\left\{\psi_{i}\right\}$, then $\psi_{j}=\psi_{i}$, and if $\psi_{i}=0$, then $\psi_{j}=0$. $\tau^{*}$ is the average transmission time of SUs and can be obtained through history observations. 

Each responsibility rating $\psi_{i}$ corresponds to a transmit power $p_{i}\;(p_{\min}\leq p_{i}\leq p_{\max})$, and the correspondence is described as the following:

(15) $$p_{i}\left(\psi_{i}\right)=\left(1-\frac{\psi_{i}}{\left|\Psi \right|}\right)p_{\min}+\frac{\psi_{i}}{\left|\Psi \right|}p_{\max}$$

where |Ψ| denotes the size of $\left\{\psi_{i}\right\}$. If SU costs a lot of time in one hop, the agent will have the responsibility to transmit the packet with larger power in the next link. It indicates how much effort the SU should make currently to be responsible for the packet transmission delay of the whole networks. The responsibility rating is adopted to help adaptively decide the transmit power of each node. It resolves the contradiction above creatively by transforming the huge action space problem to a problem with large state space, which can provide new ideas for managing the problem with large action space. We assume that there are N nodes, K channels and M levels of transmission power. The scale of network and the levels of power are huge, so that the value of both N and M is large. If the responsibility rating is not adopted and the transmission power is set as action, that is, the network state at time slot *t* is denoted as $s_{t}=\left\{n_{i}\right\}$ and the candidate actions of the $SU_{i}$ node at time slot *t* is $a_{t}=\left\{n_{j},c_{i},p_{i}\right\}$, then the size of action space is N×K×M and the size of state space is N. We can see that the size of action space will be particularly large. However, if the responsibility rating is adopted, the network state at time slot *t* is denoted as $s_{t}=\left\{n_{i},\psi_{i}\right\}$. The candidate actions of the $SU_{i}$ node at time slot *t* is $a_{t}=\left\{n_{j},c_{i},p_{\psi_{i}}\right\}$, where $p_{\psi_{i}}$ is a fixed value corresponding to $\psi_{i}$. So the size of action space is compressed to only N×K×1 and the size of state space is N×M. We can see the size of state space has risen M times and become particularly large. Therefore, the responsibility rating transforms the huge action space problem to a problem with large state space. However, the premise of responsibility rating is that SU nodes have to increase the transmission power in the next hop in order to reduce the end-to-end delay if much time is wasted in one hop transmission. So the after effects to do so is that we cannot select the levels of transmission power arbitrarily, and the level of power is determined by the transmission latency of the previous hop.

### 4.2. Problem Formulation

In this section, the joint routing and resource allocation problem is modeled as a MDP. The MDP can be represented as a tuple (S,A,T,R), where S denotes the set of environment states; A is the set of candidate actions at each state; $T=\left\{P_{s,s^{\prime }}\left(a\right)\right\}$ is the set of state transition probabilities, where $P_{s,s^{\prime }}\left(a\right)$ is the state transition probability from state s to $s^{\prime }$ when taking action a in state s; and R:S×A↦ℜ specifies the reward at s∈S when taking action a∈A. It is necessary to identify the states of SU nodes, actions of the agent and the associate rewards:

(1) States

For a given $SU_{i}$ node, the network state at time slot *t* is denoted as $s_{t}=\left\{n_{i},\psi_{i}\right\}$, where $n_{i}\in \left\{0,1,\ldots ,N-1\right\}$ represents the index of the current SU node and $\psi_{i}$ is the responsibility rating of $SU_{i}$. Since we consider a large-scale CRN with a great deal of transmission power levels, the size of state space is particularly large. 

(2) Actions

A cross-layer scheme is studied that jointly addresses routing, spectrum access and power assignment problem in CRN. We denote $a_{t}=\left\{n_{j},\;c_{i},p_{\psi_{i}}\right\}\in A$ as candidate actions of the $SU_{i}$ node at time slot *t*, where $n_{j}\in J\subset \left\{0,1,\ldots ,N-1\right\}$ is the selection of next-hop SU node, and J is the neighboring nodes set of $n_{i}\cdot c_{i}\in C_{i}$ represents the operating channel, $p_{\psi_{i}}$ denotes the transmit power described in (14). Since the size of J and $C_{i}$ is relatively small, and $p_{\psi_{i}}$ is a fixed value corresponding to $\psi_{i}$, the action space is limited for each SU.

(3) Rewards

Our objective is to reduce the end-to-end delay while improving the energy efficiency in multi-hop CRN. $\tau_{i}$ in (8) represents one-hop transmission delay, and the Power Consumption Ratio (PCR) is proposed to describe the power efficiency:

(16) $$\varepsilon_{i}=p_{\psi_{i}}/W\log_{2}\left(1+\frac{h_{ijc}p_{\psi_{i}}}{\sigma +\phi_{ijc}^{PU}}\right)$$

PCR denotes the energy consumption when getting per unit throughput. The reward $r_{t}$ assesses the immediate return incurred due to the assignment of action $a_{t}$ at state $s_{t}$. The considered reward function is:

(17) $$r_{t}=-\left(\tau_{i}+\alpha \varepsilon_{i}\right)$$

where α is the proportional coefficient to make $\tau_{i}$ coincide with order of magnitude in $\varepsilon_{i}$. Our algorithm aims to maximize this reward so that the end-to-end delay and power consumption are below the thresholds.

$n_{i}$ is the index of current SU nodes. It is only decided by the previous SU nodes and is irrelevant to the historical nodes. In addition, the current responsibility rating is relevant to the previous $\psi_{i}$ as shown in (14) and has nothing to do with the historical states. So the joint design problem can be modeled as a Markov process. Furthermore, the model-free reinforcement learning is applied in this paper, in which an accurate model for the environment is unnecessary, so that it is unnecessary to calculate the state transition probabilities. We take a minor example consisting three SU nodes and two levels of transmission power. The state transition diagram is shown as following.

**Figure 3:**
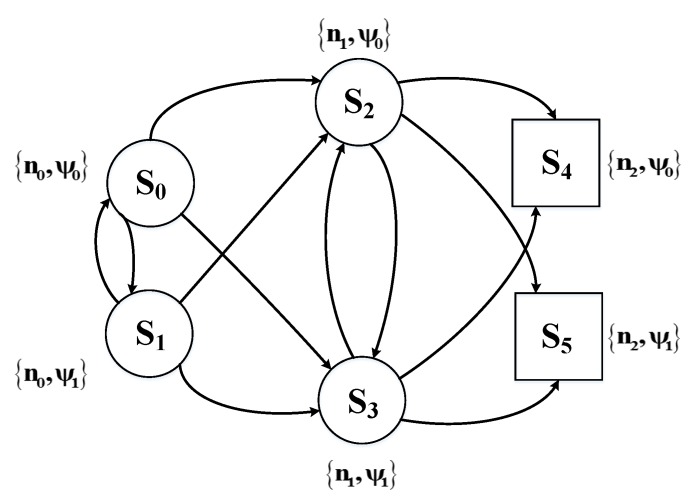
State Transition Diagram of the Markov Decision Process.

Figure 3 shows a simplest CRN containing three nodes. $n_{0}$ is the source node, $n_{1}$ is the relay node and $n_{2}$ is the destination node. $s_{4}$ and $s_{5}$ are the terminal states.

## 5. PM-DQN Based Joint Routing and Resource Allocation Algorithm

To solve the cross-layer design problem with large state space, deep reinforcement learning approach is adopted. Traditional deep reinforcement learning algorithm uses experience replay to break the temporal correlations. In order to reduce the size of replay memory while guaranteeing system performance, the Prioritized Memories Deep Q-Network is proposed in Section 5.1. Then in Section 5.2, PM-DQN is applied to the routing and resource allocation problem for minimizing the transmission delay and power consumption.

### 5.1. Prioritized Memories Deep Q-Network

DQN is adopted to solve the joint design problem because of its huge state space. One of the significant features for DQN is experience replay. The system maintains a replay memory and samples transitions from the memory to update weights of neural network. Using a replay memory leads to design choices at two levels: which experience to store, and which experience to replay. Prioritized Experience Replay addresses only the latter: replaying important transitions more frequently, and therefore learning more efficiently [25]. However, maintaining a replay memory in multi-hop CRN increases storage requirement and communication overhead. Consideration about which transitions to store and when to erase them has become an urgent problem.

**Figure 4:**
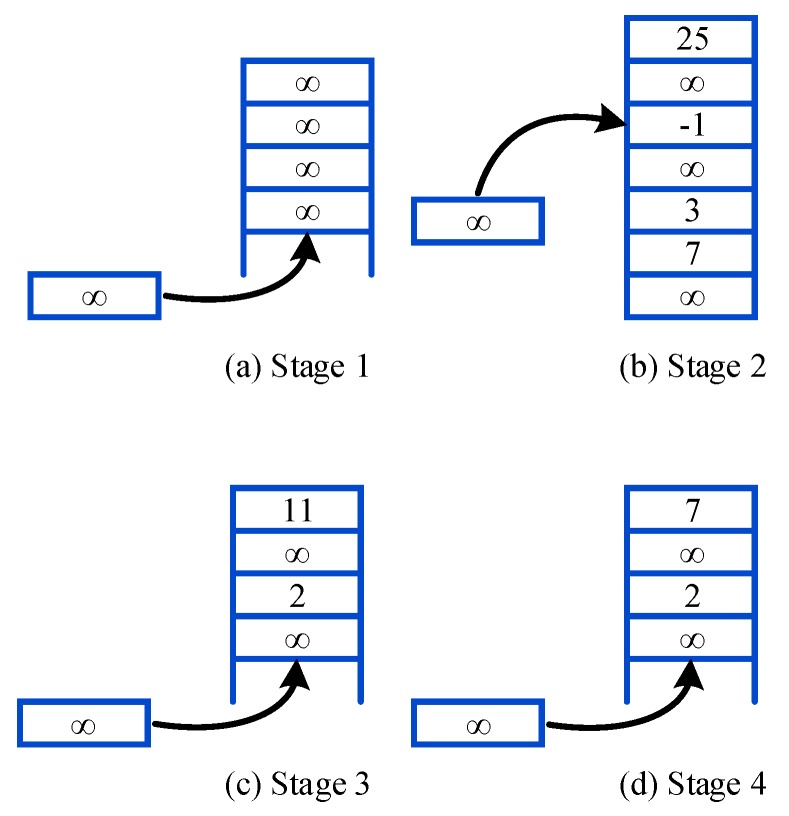
Implementation of Prioritized Memories.

To reduce replay memory and communication overhead in CRN, the Prioritized Memories Deep Q-Network is proposed. It is able to reduce the redundancy by biasing the contents of the memory to where the errors remain high. The magnitude of a transition’s TD-error δ is used as a reasonable proxy because it can indicate how far the value is from its next-step bootstrap estimate [26] and how unexpected the transition is. Specifically, the transitions whose absolute TD-errors are below a certain value will be erased, i.e., transitions with high absolute TD-errors can be retained. The implementation is demonstrated in Figure 4.

First, the format of experience at time step *t* is set as $e_{t}=\left(s_{t},a_{t},r_{t},s_{t+1},\delta_{t}\right)$, where $\delta_{t}$ represents the TD-error of the transition. It is assumed that $\delta_{t}=\infty$ before the transition is replayed, and the true TD-error is assigned to $\delta_{t}$ after the experience is sampled. If time steps are less than memory size, it means that the learning process has not started. As shown in Figure 4a, the transition is stored following the previous one, and the number of experience is increasing. In the second stage, time steps exceed memory size and the number of replayed transitions is less than a% of the memory size. The replayed experience with the lowest absolute TD-error is replaced by the new experience as shown in Figure 4b. In this period, the number of total experiences remains the same. Then we define the Erasing Critical Value (ECV) as:

(18) $${\mathrm{ECV}}_{t}=rand\left(0,\lambda \right)*\frac{1}{K_{i}}\sum_{n=0}^{K_{i}}\left|\delta_{n}\right|$$

The ECV is obtained by multiplying a random value in Λ=(0,λ),0<λ<1 with the average absolute TD-error of replayed experiences. If the number of replayed transitions is greater than a% of the memory size, the experience whose $\left|\delta_{t}\right|$ is less than the ECV will be erased. The number of experiences decreases in this stage. Fourth, if the number of replayed transitions is below b%(b<a) of the memory size, we stop erasing implementation to recover the amount of experiences, and the number of transitions increases gradually. However, the performance of PM-DQN will decrease if the value of b is too low. If the difference between a and b is too small, it is inefficient in saving system memory. So the value of a,b in this paper is set to 90 and 60 respectively, which proves to be appropriate in the simulation part. The new experience is stored following the previous one as shown in Figure 4c,d, and the agent will perform the third and the fourth step until learning process is end.

**Figure 5:**
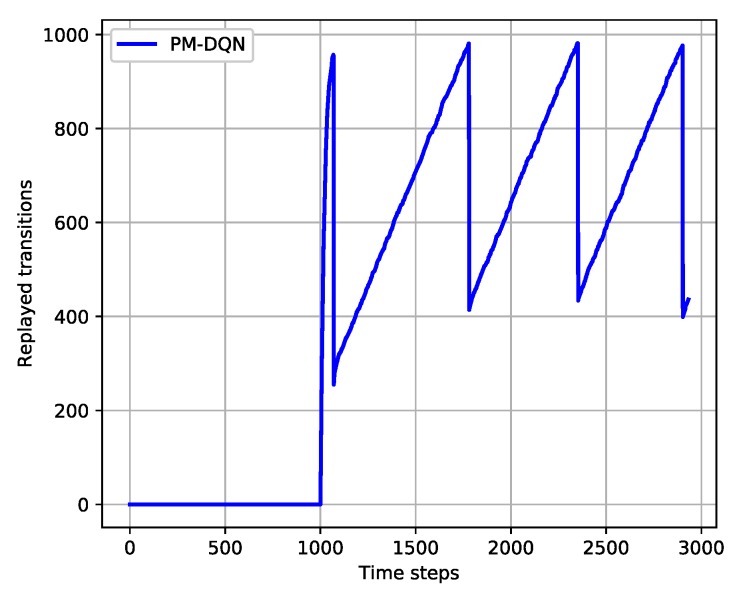
Variation trends of the transition quantities in PM-DQN.

Figure 5 shows the variation trends of transition quantities in the whole learning process of PM-DQN. We can see that the total number of the transitions fluctuates between b% and a% of the memory size. The dynamic change of the transition quantities saves the system memory while guaranteeing the performance. The details of PM-DQN are obtained as described in Algorithm 1. 

**Algorithm 1:** Prioritized Memories Deep Q-learning1: **Input:** mini-batch k, step-size η, replay period C, memory size N and erasing parameters a,b,λ 2: Initialize replay memory D=ϕ and the number of replayed experience $K_{0}=0$ 3: Initialize Q function with random weights θ and target $\hat{Q}$ function with weights $\theta^{-}=\theta$ 4: **For**
eposide=1,M
**do** 5: Initialize state $s_{1}$ 6: **For**
t=1,T
**do** 7:  With probability ε select a random action $a_{t}$ 8:  otherwise select $a_{t}=\arg\max_{a}Q\left(s_{t},a;\theta \right)$ 9:  Execute action $a_{t}$ and observe reward $r_{t}$, state $s_{t+1}$ 10:  Store transition $\left(s_{t},a_{t},r_{t},s_{t+1},\delta_{t}\right)$ in D 11:  Sample mini-batch of transitions $\left(s_{i},a_{i},r_{i},s_{i+1},\delta_{i}\right)$ from D 12:  Set $y_{i}=\left\{ \begin{array}{l} r_{i}\;\mathrm{if}\;\mathrm{episode}\;\mathrm{terminates}\;\mathrm{at}\;\mathrm{step}\;i\;+1\\ r_{i}+\gamma \max_{a^{\prime }}\hat{Q}\left(s_{i+1},a^{\prime };\theta^{-}\right)\;\mathrm{otherwise} \end{array}\right.$ 13:  Perform gradient descent on ${\left(y_{i}-Q\left(s_{i},a_{i};\theta \right)\right)}^{2}$ with respect to the network parameters θ 14:  Compute ${\mathrm{ECV}}_{t}=rand\left(0,\lambda \right)*\frac{1}{K_{i}}\sum_{n=0}^{K_{i}}\left|\delta_{n}\right|$ 15:  **If**
$\left(K_{t}>a\%*N\right)\;or\;\left(\left(K_{t}<K_{t-1})\;and\;(K_{t}\geq b\%*N\right)\right)$
**do** 16:   **For**
transition=1,N
**do** 17:    **If**
$\left|\delta_{transition}\right|<{\mathrm{ECV}}_{t}$
**do** 18:     erase the transition 19:   **End For** 20:  Every C steps reset $\hat{Q}=Q$ 21: **End For** 22: **End For**

### 5.2. PM-DQN-Based Joint Routing and Resource Allocation Algorithm

In order to solve the cross-layer design problem with large state space and save the system memory, PM-DQN is applied to the routing and resource allocation problem. The joint design scheme requires merely the source node $n_{0}$ to equip the RL agent and maintain replay memories. It enormously reduces the communication overhead while guaranteeing system performance.

**Figure 6:**
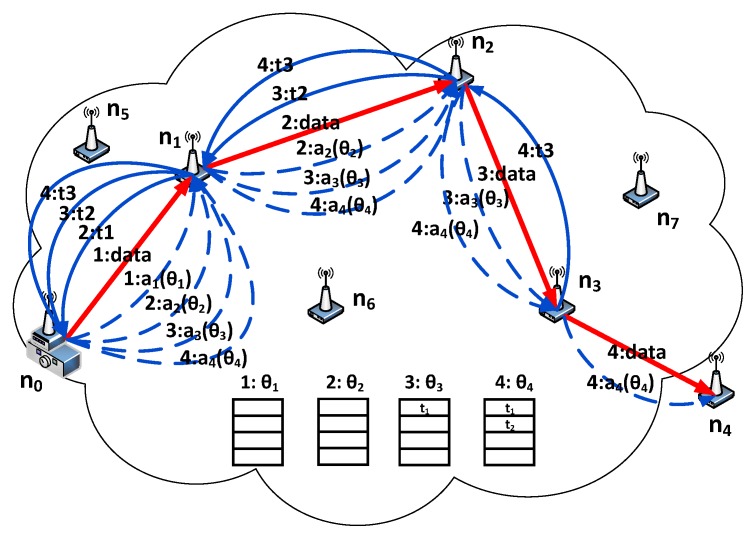
Transmission mechanism of replay memory in joint design scheme.

The mechanism of PM-DQN based joint routing and resource allocation scheme is shown in Figure 6, and the serial number in Figure 6 denotes the order of stage. Initially, the agent in the source node $n_{0}$ uses the initial weights of neural network $\theta_{0}$ to compute Q value and chooses $n_{1}$ as its next hop. In the first stage, $n_{0}$ performs data transmission. Before every data transmission, the SU node broadcasts a relay request (RREQ) message using the CCC which includes its node ID. Upon receiving the RREQ, the neighboring nodes which are closer to the destination send back the relay reply (RREP) messages. The neighbor information in RREP will be back propagated with the newest transition to the source node in the subsequent stages, so that the agent can identify unavailable actions during weights updating of neural network. As $n_{0}$ transmits data using DTC, the agent updates the weights of neural network to $\theta_{1}$ by sampling transitions from the previous memories and then selects the action $a_{1}$ with probability [27]:

(19) $$\pi \left(s_{t},a_{t}\right)=\frac{e^{Q\left(s_{t},a\right)/\eta }}{\sum_{b\in A}e^{Q\left(s_{t},b\right)/\eta }}$$

where η is a positive parameter called the temperature. High temperature causes the action probabilities to be all nearly equal, and low temperature causes huge difference in selection probabilities for actions. After that the action $a_{1}$ is transmitted to SU node $n_{1}$ through CCC. After the data transmission is finished, node $n_{1}$ obtains the reward and generates the newest transition $t_{1}$. 

In the second stage, $n_{1}$ selects $n_{2}$ as its next hop according to action $a_{1}$ and performs data transmission using DTC. Then the newest transition $t_{1}$ is back propagated to the source node through CCC. At the same time, the agent updates the weights to $\theta_{2}$ by sampling transitions from the same memories as the first stage and selects the action $a_{2}$ of $n_{2}$ using weights $\theta_{2}$. If the selected action is available, i.e., the next relay $n_{j}$ is in the neighboring set of the current node, the target Q value is set to $r_{i}+\gamma \max_{a^{\prime }}\hat{Q}\left(s_{i+1},a^{\prime };\theta^{-}\right)$ in the weights updating stage of neural network; otherwise, the target Q value is set to a certain negative value. After that the action $a_{2}$ is transmitted to SU node $n_{2}$. That is, the SU node performs data transmission through DTC and transmits signaling such as transitions and actions using CCC in the meanwhile. Since the size of one transition or action is particularly small, the transition or action can be successfully transmitted before the data transmission is over and will not cause additional transmission delay. Also, node $n_{2}$ obtains the reward and generates the newest transition $t_{2}$ when the data transmission is over. The transmission process of data and signaling in the following stage is similar, and the only difference is that the replay memory has been updated step by step. As shown in Figure 6, in the third stage the memories comprise transition $t_{1}$, and transition $t_{1}$ with $t_{2}$ in the fourth, and so on. The details of Centralized Joint Routing and Resource Allocation Algorithm are obtained as described in Algorithm 2.

**Algorithm 2:** PM-DQN Based Joint Routing and Resource Allocation Algorithm1: **Input:** mini-batch k, step-size η, replay period C and memory size N 2: Initialize replay memory D=ϕ and the number of replayed experience $K_{0}=0$ 3: Initialize Q function with random weights θ and target $\hat{Q}$ function with weights $\theta^{-}=\theta$ 4: **For**
eposide=1,M
**do** 5: Initialize state $s_{1}=\left\{n_{1},\;\psi_{1}\right\}$ 6: **For**
t=1,T
**do** 7:  Node $n_{i}$ selects next relay $n_{j}$ according to $a_{t}$ which is obtained from the former stage 8:  Observe reward $r_{t-1}$ and generate the newest transition $\left(s_{t-1},a_{t-1},r_{t-1},s_{t},\delta_{t-1}\right)$ 9:  Node $n_{0}$ samples mini-batch of transitions $\left(s_{k},a_{k},r_{k},s_{k+1},\delta_{k}\right)$ to update weights 10:  Set $y_{k}=\left\{ \begin{array}{l} -m(m>0)\;\mathrm{if}\;\mathrm{the}\;\mathrm{selected}\;\mathrm{action}\;\mathrm{is}\;\mathrm{unavailable}\\ r_{k}+\gamma \max_{a^{\prime }}\hat{Q}\left(s_{k+1},a^{\prime };\theta^{-}\right)\;\mathrm{otherwise} \end{array}\right.$ 11:  Perform gradient descent on ${\left(y_{k}-Q\left(s_{k},a_{k};\theta_{i}\right)\right)}^{2}$ and select action $a_{t+1}$ of the next relay $n_{j}$ according to (19) 12:  Execute action $a_{t}=\left\{n_{j},c_{i},p_{\psi_{i}}\right\}$: Transmit data to $n_{j}$ through $c_{i}$; meanwhile transmit $a_{t+1}$ to $n_{j}$  and send $\left(s_{t-1},a_{t-1},r_{t-1},s_{t},\delta_{t-1}\right)$ back to $n_{0}$ 13:  Do Prioritized Memories Algorithm in $n_{0}$ 14:  Every C steps reset $\hat{Q}=Q$ 15: **End For** 16: **End For**

## 6. Simulation Results

In this section, we evaluate the performance of our joint routing and resource allocation scheme. The results of the proposed PM-DQN based joint design algorithm is compared with (i) Q-routing; (ii) DQN based scheme; and (iii) Fixed Power Q-routing (FP-Q-routing) [15] which does not consider power allocation and its transmission power is fixed. We set the bandwidth of DTC to be W=1 MHz, and the bandwidth of CCC is 2 MHz. It is supposed that the AWGN power $\sigma =10^{-7}\;\mathrm{mW}$, the packet size is $2\times 10^{5}\;\mathrm{bit}$, and the link gain used in this paper is given by:

(20) $$h=LF{\left(\frac{d}{d_{0}}\right)}^{-n},\;\mathrm{for}\;d>d_{0}$$

where L is a constant set to be $10^{-6}$, and the shadowing factor F is subject to a lognormal distribution with a mean of 0 dB and variance of 6 dB, d is the actual distance between the transmitter and receiver, $d_{0}$ is the reference distance, and n is the path loss exponent. In our simulation process, we set $d_{0}=1$ and n=4. To compare the performance of the algorithms, we consider two different kinds of experimental scenarios varying in the number of SU nodes, PU channels and transmission power levels. In the first experiment, a CRN with 10 SU nodes and 5 PU channels deployed in a 300 m × 300 m region is considered, and the discrete transmission power of SU nodes is divided into 5 levels: {50, 100,…,250 mW}. The size of state space is calculated as 10×5=50. In the second experiment, a CRN consisting of 50 SU nodes and 10 PUs uniformly distributed over a 1000 m × 1000 m square area is considered, and the transmission power is divided into 10 levels: {50, 100,…,500 mW}. The size of state space is 50×10=500 which is ten times larger than it in the first experiment. The expected mean and deviation of average PU departure rate $\theta_{d}(\mu ,\sigma )$ are set as μ=0.1 and σ=0.05. In addition, we set the erasing parameters a, b, λ to 90, 60, 0.5 respectively.

### 6.1. Overall Performance of Routing Schemes

**Figure 7:**
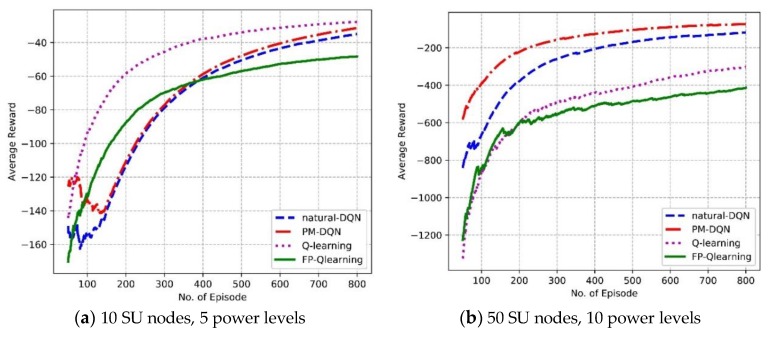
Average reward of the SU agent vs. the number of episodes.

We first evaluate the overall performance of average reward and convergence property in different experiment scenarios. Figure 7 shows the average reward the SU agent gets as a function considering the number of episodes. It is found that the average reward increases with increasing number of episodes for all algorithms, which means that the agent gets more scores as interacts with the environment and gradually learns the characters of it. In Figure 7a, it is found that Q-routing achieves the highest average reward, and the reward of PM-DQN based scheme is slightly higher than natural DQN. This is mainly because that state space of the network is relatively small and the training data is scarce. It is difficult to fully adjust the parameters of neural network so that Q-routing has a slight advantage over DQN methods. In addition, scarce samples make little difference between prioritized memories and original memories so that the performance of PM-DQN based algorithm is close to that of natural DQN. In Figure 7b, it is observed that the reward of PM-DQN based approach is more superior to natural DQN scheme, and both DQN based methods have obvious advantages comparing to Q-routing and FP-Q-routing. This is because large state space provides huge amounts of training data, thus the neural network and prioritized memories can work effectively. However, FP-Q-routing obtains the lowest average reward in both Figure 7a,b, which illustrates the importance of power allocation. So the metric of responsibility rating can improve the system performance distinctly.

**Figure 8:**
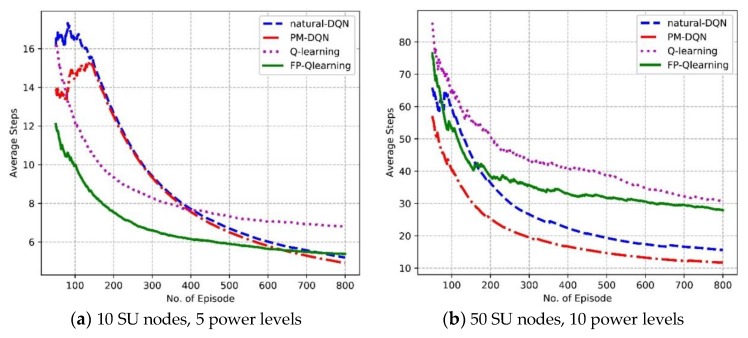
Average steps vs. the number of episodes.

Figure 8 shows the convergence properties of four protocols in different kinds of experimental environments. Figure 8a illustrates that with increasing number of episodes, the average steps suffers a gradual decline for all algorithms in the network with 10 SU nodes and five power levels. The convergence speed of Q-routing and FP-Q-routing is faster than two DQN-based algorithms before 400 episodes. After 400 episodes, the Q-routing stabilizes at seven steps, and the average steps of two DQN based protocols continue to fall and converge to five steps. This is because the neural network has not been trained well at first so that the DQN methods converge slowly. However, in the later period the benefits of experience replay become apparent. It makes DQN based schemes choose links with high rewards since old transitions will be stored and reused to exploit the training data deeper. Thanks to the small scale of the network and limited training data, the performance of PM-DQN based scheme is similar to the DQN method. In addition, the average steps of FP-Q-routing are less than Q-routing. This is because that responsibility rating is not adopted in FP-Q-routing so that the demission of states is relatively small and extra steps will not be expensed to allocate transmission power. In Figure 8b, the DQN based methods converge much faster than Q-routing and FP-Q-routing, and the scheme based on PM-DQN achieves less average steps than natural DQN. This results from the advantage of neural network in huge state space. Furthermore, prioritized memories with sufficient samples contribute more valuable transitions so that the convergence speed of PM-DQN based method is faster than the natural DQN.

### 6.2. Performance of Effectiveness

**Figure 9:**
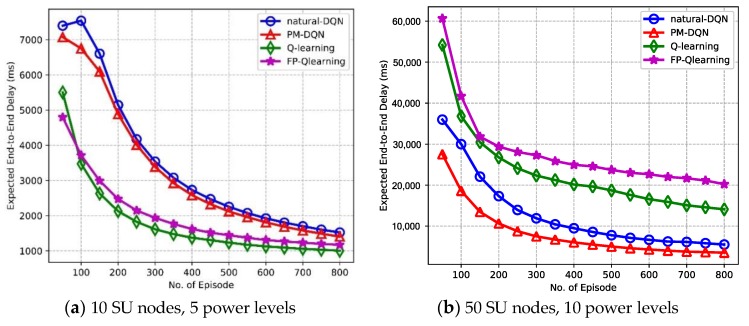
Expected end-to-end delay vs. the number of episodes.

The effectiveness properties of the end-to-end delay and power consumption efficiency are investigated in this section. As shown in Figure 9, it is observed that the expected end-to-end delay degrades with increasing number of episodes. Figure 9a compares the delay performance for four kinds of protocols in the network with 10 SU nodes. It is found that the end-to-end delay of Q-routing and FP-Q-routing suffers a sharply drop in the beginning, and it retains almost steady state after about 400 episodes. While the delay of two DQN based schemes falls slowly before 400 episodes, and after that the convergence speed becomes faster than Q-routing and FP-Q-routing. The performance of four protocols is almost the same in the end. As shown in Figure 8a, the convergence speed of Q-routing and FP-Q-routing is faster than DQN-based schemes before 400 episodes, and then the curves become flatter, which results that the delay of Q-routing and FP-Q-routing falls faster than it of two DQN based algorithms at first but loses ground in the end. In addition, the latency of FP-Q-routing is longer than Q-routing because FP-Q-routing fails to transmit data with high power when the communication condition is better so that it has longer time delay in total. Figure 9b shows that the performance of natural DQN based method is superior to Q-routing and FP-Q-routing in the network with 50 SU nodes, and PM-DQN achieves the least end-to-end delay. It illustrates the benefits of deep Q-learning and prioritized memories method in the network with large state space.

**Figure 10:**
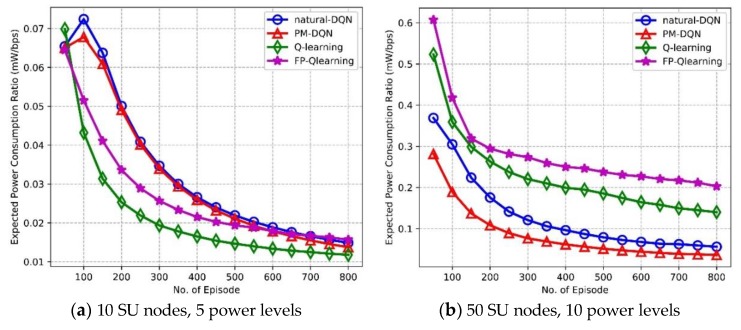
Expected power consumption ratio vs. the number of episodes.

Figure 10 depicts expected PCR as a function of amount of episodes. With the increasing number of episodes, the trend of PCR is very similar to that of end-to-end delay shown in Figure 9. That is, in Figure 10a the PCR of Q-routing and FP-Q-routing falls sharply in the beginning, is seemingly steady after 400 episodes, and achieves almost the same performance as DQN-based methods in the end. In addition, the PCR of FP-Q-routing is larger than Q-routing because FP-Q-routing fails to adjust transmission power adaptively and it may still transmit data with high power in poor communication conditions, which causes very low energy efficiency. In Figure 10b, energy efficiency of DQN-based methods is significantly improved comparing to Q-routing and FP-Q-routing. The PM-DQN-based scheme achieves the least PCR, which is 25% less than it of natural DQN. Consequently, in the small scale network PCR performance of four protocols is relatively close. Furthermore, in the network with large state space, DQN based methods consume less power comparing to Q-routing and FP-Q-routing, and prioritized memories experience replay further enhances the power efficiency.

### 6.3. Reliability Properties

**Figure 11:**
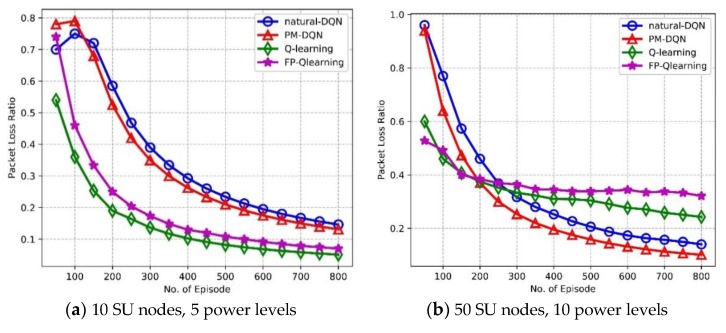
Packet loss ratio vs. the number of episodes.

The reliability and robustness are significant properties in the communication networks. We further compare the performance of PM-DQN with that of DQN, Q-routing and FP-Q-routing to evaluate the Packet Loss Ratio (PLR) and estimation error. Figure 11a shows the trend of PLR as a function of episodes in the network with 10 SU nodes. PLR declines as the learning process goes on, and Q-routing achieves the lowest PLR, followed by FP-Q-routing. PM-DQN based scheme holds a slight advantage over natural DQN. The reason for this is the same as it in Figure 9a. Comparison of PLR in the network with 50 SU nodes is shown in Figure 11b. PLR of Q-routing and FP-Q-routing is much lower than two DQN based schemes in the beginning, and after about 200 episodes PLR of PM-DQN based scheme achieves the least and keeps the advantage till the end. While after around 300 episodes, PLR of DQN based scheme is lower than Q-routing and FP-Q-routing. This is due to the fact that, since at first the end-to-end delay of Q-routing and FP-Q-routing falls faster than it of two DQN based algorithms as shown in Figure 9b, the total number of episodes whose routing latency exceeds the threshold is less than it of DQN based methods so that its PLR is relatively low. However, the delay of Q-routing and FP-Q-routing drops slightly after that and therefore its PLR is higher than it of the other two schemes.

**Figure 12:**
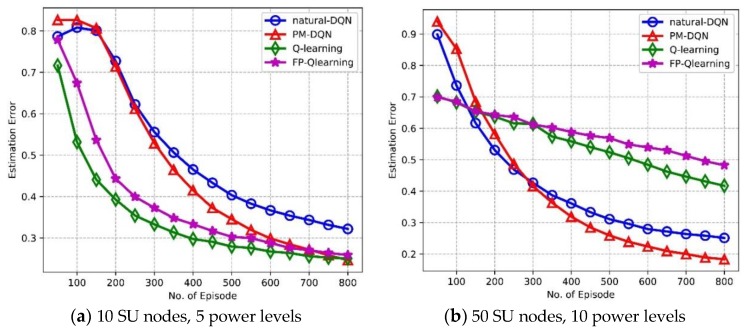
Estimation error vs. the number of episodes.

In the learning process the agent may select some invalid actions such as non-neighbor nodes or unavailable channels. Estimation error denotes the proportion of steps choosing invalid actions in the total time steps. It is observed from Figure 12a,b that PM-DQN based routing scheme achieves the least estimation error in the network with either 10 or 50 SU nodes. The primary reason for this is that the invalid actions result in very low target Q value (we set it to –100 in the simulation) corresponding to relatively large TD-error. In PM-DQN, the prioritized memories preserve most of these transitions so that the probability of selecting the invalid actions reduces sharply even in the small scale network. In Figure 12a, we can see that estimation error of Q-routing and FP-Q-routing is much lower than it of DQN and is almost the same as PM-DQN method. However, estimation error of Q-routing and FP-Q-routing is significantly high in network with 50 nodes as shown in Figure 12b. Network with larger state space results in more invalid actions. In Q-routing and FP-Q-routing, the current parameters determine the next data sample, which could make the parameters get stuck in a poor local minimum in large state space. This is what leads to the trend of the curves.

### 6.4. Effect of Learning Rate and Q-value

**Figure 13:**
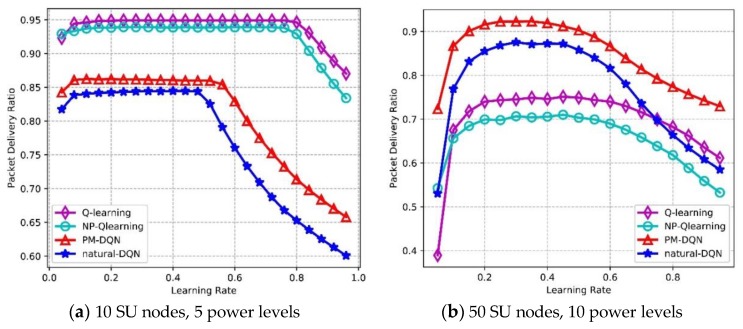
Packet delivery ratio vs. learning rate.

Figure 13 depicts the effects of learning rate on Packet Delivery Ratio (PDR). In Figure 13a, it is found that the PDR of four algorithms is flat in the beginning, then the PDR of PM-DQN and DQN based schemes drops sharply after learning rate reaches 0.5, and the PDR of Q-routing and FP-Q-routing (i.e., Non-Power Adaption based Q-learning, NP-Q-learning) decreases when learning rate is 0.8. This is because Q-value of the selected actions will become particularly large if learning rate is high according to (11). Larger Q-value leads to higher probability of choosing these actions again, so that the opportunity of exploring other actions will be lower and the performance decreases rapidly. In the network with 10 SU nodes, Q-routing achieves the highest average PDR, and DQN has the lowest PDR. In Figure 13b, it is observed that the PDR of PM-DQN and DQN based schemes is unimodal curve, which reaches the maximum value when learning rate is 0.3, and then decreases distinctly. The PDR of Q-routing and FP-Q-routing rises before the learning rate reaches 0.4, and after that the curves drop gradually. In the second experiment, PM-DQN achieves the highest PDR, followed by DQN. The PDR of Q-routing is lower than DQN, and FP-Q-routing obtains the lowest PDR. In addition, the weights adjustment of neural network is more sensitive to learning rate than the update of Q-learning, so that the PDR of two DQN based schemes changes more apparently with the increasing of learning rate than Q-routing and FP-Q-routing.

**Figure 14:**
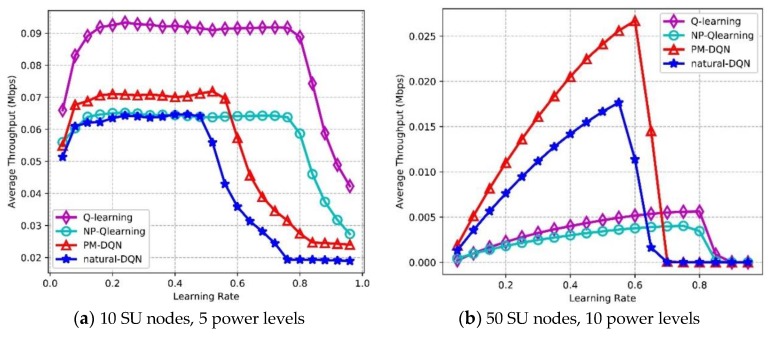
Average throughput vs. learning rate.

Figure 14 shows the effects of learning rate on average throughput. In Figure 14a, the throughput of PM-DQN and DQN based schemes is almost flat at first, and then the throughput declines sharply after learning rate is 0.5. The throughput of Q-routing and FP-Q-routing is also flat before learning rate reaches 0.8, and after that a sharp decline is on the curve. Q-routing achieves the highest average throughput, and the throughput of PM-DQN is much lower than Q-routing. Both DQN and FP-Q-routing obtain the lowest throughput. In Figure 14b, the throughput of PM-DQN- and DQN-based schemes increases at first, and achieves the highest throughput when learning rate is around 0.55. After that, the throughput declines sharply. The throughput of Q-routing and FP-Q-routing has similar trend as two DQN based methods and obtains the highest throughput at 0.8. The reason for this is that the update of Q-value is slow with small learning rate so that the learning efficiency is low at first. However, when learning rate gets particularly large, the agent will only exploit the selected actions and fail to explore other actions, which also results in performance degradation. In the large-scale network, PM-DQN achieves the highest average throughput, and the throughput of FP-Q-routing is the lowest. Furthermore, it is found that the throughput of two DQN based algorithms also changes more obviously than Q-learning based schemes with the increasing of learning rate.

**Figure 15:**
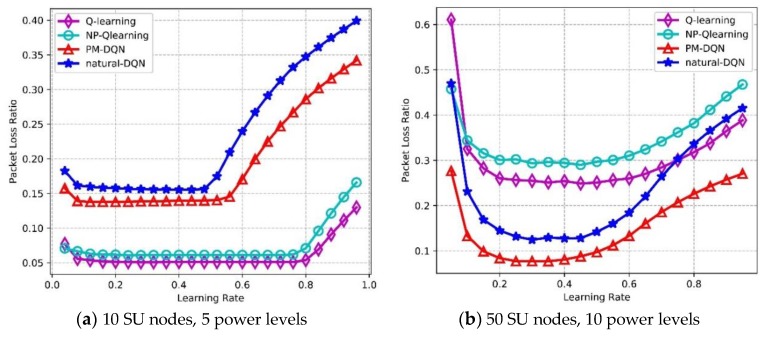
Packet loss ratio vs. learning rate.

Figure 15 depicts Packet Loss Ratio (PLR) as a function of learning rate. PLR reflects routing stability of the network, and lower PLR means less loss of transmission data as well as higher reliability. In the network with 10 SU nodes, the PLR of four protocols is flat at first, while the PLR of two DQN based methods increases rapidly after learning rate is 0.5, and the curves of Q-routing and FP-Q-routing rise at around 0.8. The PLR of Q-routing is the lowest, and DQN based method has the highest PLR. In Figure 15b, the curves of four algorithms are unimodal. The PLR of two DQN based methods reaches the minimum value when learning rate is 0.3, and then it rises distinctly. When learning rate reaches 0.4, Q-routing and FP-Q-routing obtain the lowest PLR. The reason for this is similar to those discussed above. In the second experiment, PM-DQN achieves the lowest PLR in four algorithms, followed by DQN. The PLR of Q-routing is much higher than DQN, and FP-Q-routing has the highest PLR.

**Figure 16:**
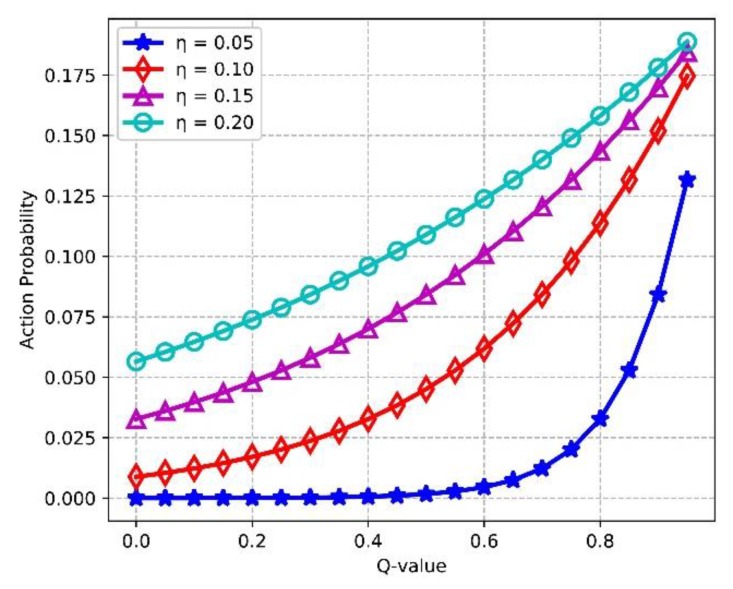
Action probability vs. Q-value.

Figure 16 shows the effect of Q values for improving the learning efficiency of the agent. We assume that the agent has 5 available actions in state $s_{t}$, and the Q values of other actions are equal to 1. It is found that with increasing of Q-value, the action probability $P\left(s_{t},a_{i}\right)$ of choosing the action $a_{i}$ in $s_{t}$ rises. Higher action probability means more opportunities of exploitation for the corresponding action, i.e., the learning efficiency of the agent is improved as Q-value grows. In addition, $P\left(s_{t},a_{i}\right)$ will be higher if the temperature η gets larger, which indicates that the learning efficiency gets enhanced with the increasing of η in (19).

### 6.5. Complexity Analysis

The number of messages exchanged in the network is analyzed as following: as illustrated in Section 5.2, the first hop of data transmission needs only 1 message exchange; The second hop needs three message exchanges including two action messages to be sent forward and one transition message to be back propagated; The third hop needs five message exchanges which comprise three action messages and two transition messages, and so on, so that the $n_{th}$ hop needs 2n−1 messages to be exchanged in total, which contains n action messages and n−1 transition messages. We assume that N SUs uniformly distribute over a square area in our experiment, which means that the average route length can be approximated to $\sqrt{N}$. So the number of message exchange can be calculated as $\left[\frac{1}{2}\left(1+\left(2\sqrt{N}-1\right)\right)\times \sqrt{N}\right]/\left(1-P_{error}\right)=N/\left(1-P_{error}\right)$, where $P_{error}$ is the average estimation error when the algorithm converges. That is, the complexity of message exchange for every route is $o\left(N/\left(1-P_{error}\right)\right)$.

Then the time complexity of the joint design algorithm is taken into consideration. In the experiment, the neural network has three layers. The number of neurons in the first and third layer is equal to the dimension of the states $n_{feature}$ and the size of action space $n_{action}$, respectively, and the number of the second layer is set to be $n_{2}$. The feed-forward calculation for one sample needs two matrix operations, which requires $n_{feature}\times n_{2}$ and $n_{2}\times n_{action}$ times of computation. So the time complexity for one sample is $o\left(n_{feature}\times n_{2}+n_{2}\times n_{action}\right)=o\left(n_{2}\right)$. We assume that the algorithm converges after K episodes. Since the average route length is approximated to $\sqrt{N}$, the average steps of one episode is $\sqrt{N}/\left(1-P_{error}\right)$, which means that the number of total samples is calculated as $K\times \sqrt{N}/\left(1-P_{error}\right)$. Therefore, the time complexity of the joint design algorithm is $o\left(n_{2}\times K\times \sqrt{N}/\left(1-P_{error}\right)\right)$.

## 7. Conclusions

In this paper, we have proposed a PM-DQN-based joint routing and resource allocation scheme for cognitive radio ad hoc networks. Simulation results show that PM-DQN-based joint design scheme reduces the memory occupancy while slightly improving system performance compared to the scheme based on natural DQN. Furthermore, both DQN-based methods reduce the average end-to-end delay, packet loss ratio and estimation error while achieving higher energy efficiency in comparison with Q-routing. In this paper, single-agent reinforcement learning with limited capacity is adopted. However, learning strategy with multiple agents is more suitable for solving complicated problem in multi-hop CRN. Our future work will aim at solving the resource management problem in CRN using multi-agent learning strategy.
